# The Role of Employee Relations in Shaping Job Satisfaction as an Element Promoting Positive Mental Health at Work in the Era of COVID-19

**DOI:** 10.3390/ijerph18041903

**Published:** 2021-02-16

**Authors:** Helena Bulińska-Stangrecka, Anna Bagieńska

**Affiliations:** 1 Faculty of Administration and Social Sciences, Warsaw University of Technology, 02-786 Warsaw, Poland; 2 Faculty of Engineering Management, Bialystok University of Technology, 15-351 Bialystok, Poland; a.bagienska@pb.edu.pl

**Keywords:** job satisfaction, employee relations, trust, COVID-19, SARS-CoV-2, well-being

## Abstract

The COVID-19 pandemic is affecting the mental health of employees. Deterioration of the well-being of workers is also caused by changes in the working environment. Remote working can affect both social interactions and job satisfaction. The purpose of the study is to examine what factors influence job satisfaction in the context of remote work caused by a pandemic. The study analyses whether employee relations and interpersonal trust are related to the level of perceived job satisfaction. The investigation started with a literature review and then research hypotheses have been formulated. Based on an empirical study, carried out on a sample of 220 IT employees during the pandemic, an analysis of the mediating role of trust in links between employee relations and perceived job satisfaction was conducted. The current study found that positive employee relations contribute to the level of job satisfaction. Additionally, trust is an important factor that mediates these relationships. Based on the results of the research, it was possible to describe the mechanism of shaping a supportive work environment during a pandemic.

## 1. Introduction

Positive mental health at work is an extremely important factor in the management of today’s organizations [1]. The promotion of positive mental health enables the improvement of the working atmosphere and is associated with a positive impact on employees and the effects of their work tasks [2]. Especially in times of sudden changes and reorganization caused by important events such as the COVID-19 pandemic, the issue of positive mental health at work is gaining importance. The emergence of the disease caused by the coronavirus SARS-CoV-2 caused a number of changes in the functioning of society and enterprises. In order to prevent the spread of the virus, a state of epidemic emergency was introduced in several countries. In Poland, from 14 March 2020, many industries have been forced to completely suspend their business activities. In schools, administration, and some enterprises, in order to maintain business continuity, rotational, hybrid or remote working was used. The use of preventive measures was necessary to reduce the transmission of the COVID-19 virus. The COVID-19 virus still poses a significant threat in Poland and around the world, and various restrictions are still in place to reduce the spread of the virus. However, these restrictions also affect organizations and employees.

Until the pandemic broke out, remote work was mostly done by highly skilled workers with a lot of autonomy who did their work via computer. In 2018 in EU countries, about 40% of IT and other communication services workers worked partially from home [3]. During the pandemic, the IT sector was one of the first to adopt remote employment. Since the outbreak of the Covid-19 pandemic, working from home has become the norm for millions of workers in the EU and around the world. Early estimates by Eurofound [4] indicate that nearly 40% of people currently working in the EU have started working full-time remotely as a result of the pandemic. The change in the way of working, and the additional workloads contribute significantly to the changing situation of the employees.

As a result of the changes caused by the pandemic, employee anxiety, stress [5,6] changes in risk perception [7,8] experiencing anxiety of isolation, stigma, discrimination [9] has increased significantly. Stress was caused by new factors such as health and life threats, numerous restrictions and recommendations due to the epidemic state (stay-at-home, closure of many institutions), isolation and lack of social support, disturbed work-home balance, lack of sufficient physical activity lowering overall stress resistance [10]. It is therefore important to understand the mechanisms supporting positive mental health beyond the safety of the working environment. 

One of the factors that contribute significantly to positive mental health is job satisfaction [11,12]. Studies to date indicate that satisfaction is a key indicator for positive mental health at work [13]. Moreover, job satisfaction comes from a subjective comparison by the individual of the actual work situation with the expected one [14]. Furthermore, it is regarded as a cornerstone of positive well-being at the workplace [15].

Specifically, this study highlights the role of employee relations in remote working conditions caused by the pandemic. In general, employee relationships reflect the positive interactions between employees in organizations [16]. With the increase in various forms of remote working (telework from home, mobile telework) [17,18,19] and the occupational stress resulting from the pandemic [3], employee relations have also been transformed. The ways of communication within the organization are changing, and so are employee relations. Since the results of study carried out by Abun, et al. [20] suggest that employee relationship influences job satisfaction among employees in academia, we assume that they will also enhance the sense of job satisfaction among IT employees during the COVID-19 pandemic. Therefore, this study focuses on a better understanding of the factors supporting the job satisfaction, and in consequence a promotion of positive mental health during the COVID-19 pandemic. The purpose of the study is to examine if employee relations and interpersonal trust influence job satisfaction in the context of remote work caused by a pandemic.

In addition, the integration of the concept of trust in this context enables an in-depth analysis of underlying interrelationships. This allows building accurate models that reflect complex organizational phenomena that can be used in practice by managers who want to promote positive mental health at work by increasing job satisfaction.

The main argument and contribution of this paper is three-fold. First, our study addresses the issue of employee mental health during the COVID-19 pandemic. Secondly, it proposes an analysis of the social factors that have an impact on employees’ psychological well-being in relation to Expectation states theory (EST). It explains how attitudes are formed based on alignment with perceptions of expectations in the group. Finally, it examines, based on empirical data, the mechanisms of the interplay between interpersonal trust, employee relations and job satisfaction to predict employee well-being.

## 2. Materials and Methods

### 2.1. Theoretical Development

The Expectation states theory (EST) is considered to be one of the interactive theories relating to cooperative activities in social groups [21]. The main premise of the theory is to assume that individuals are in a task relationship with each other and, on the basis of the available information, formulate expectations for the actions of others in relation to specific tasks [22]. The behavior of the individual, on the other hand, is the origin of his or her expectations. Moreover, the behaviors reinforce existing expectations.

An important element of EST is the assumption that states of expectations have an episodic nature and become active when a given structure stabilizes. Changes in the environment affect the available information and, as a result, lead to changes in the states of expectation, and thus relate to a certain renegotiation of reality in response to the emerging external condition [23]. EST explains the issues of social influence in working groups and the role of interactions of team members on their behavior [24].

EST refers to groups with a collective goal-oriented approach (for instance in the context of work or school). Collective action implies the formation of expectations about the anticipated behavior of other group members. The behavior of group members is then shaped based on these expectations. As a result, patterns of interchangeable behavior are established within the group. For instance, an employee who demonstrates a contribution to group activities gets more chances to perform. With the increased credibility received from group members and the derived greater scope for action, his/her well-being in the group improves. In other words, positive expectations of the individual lead to specific positive group behavior. Thus, having good relationships with colleagues, leads to the development of positive expectations, subsequently triggering positive group attitudes, leading to higher satisfaction with participation in the group [24].

Enabling the exchange of information and providing opportunities for interaction to build positive expectations for employees at work is an important element in ensuring positive group processes during remote work. Both the feedback and support from colleagues on the tasks performed in a remote working setting are the driving force behind group processes in a team.

In the context of organization management, EST provides a theoretical framework to conceptualize the factors that contribute to job satisfaction based on information from the surrounding environment [25]. EST indicates how performance expectations about others are constructed within the organization based on informational clues [26]. Indeed, it helps to understand the process of constructing expectations, which determines the behavior of task force members. Based on EST, it becomes possible to explain how employee relationships affect job satisfaction. The interactions and the associated expectation-related consequences are formed as a result of activating events (work task). Following the analysis of available informational clues (relations with co-workers), individuals’ performance expectations are formulated. Next, individuals behave according to their expectations in a given context. Performance expectations are, in this respect, stable structures that determine the relationship between the participants of an interaction [organization] and influence the development of subsequent expectations [27]. In accordance with EST, job satisfaction is the result of an individual’s expectations of his or her co-workers, established on the basis of the information available in a given context. Consistent with EST, trust in managers, especially with regard to his or her ability to carry out future tasks, fits perfectly into the dynamics of these relationships based on the performance expectation. Thus, EST contributes to a better understanding of these analyzed interrelations.

### 2.2. Employee Relations and Job Satisfaction

Work satisfaction is most often defined as the positive emotional state resulting from the employee’s professional experience [28] or the degree of employees’ contentment with their work [29]. Overall job satisfaction can be seen as a structure that shapes and aggregates satisfaction with certain aspects of work [30]. Job satisfaction and non-work related satisfaction are seen as two different concepts that explain work-life balance [31]. Job satisfaction, as one of the aspects of well-being, is thus considered to be a positive attribute of mental health [32].

The significance of job satisfaction for both the employee and the organization means that it is still the subject of many studies. The surveys on job satisfaction include the ways of measuring it [33], its impact on engagement [34], productivity [35], company performance [36,37,38], intention to change employment [39]. Research on remote work indicates that temporarily performing duties away from the workplace can increase employee job satisfaction [40]. In contrast, research conducted under social isolation and COVID stress suggested that isolation negatively affects remote job satisfaction [41].

Job satisfaction is an extremely complex concept, influenced by various factors and their groups. Therefore, job satisfaction is a key factor in the context of the efficient functioning of contemporary organizations. For this reason, a central aspect of the organization’s research are the factors that affect employee satisfaction in the workplace. Satisfaction can be considered at both organizational and individual level. The organizational factors include remuneration, promotion opportunities, communication with superiors and benefits offered to employees. Individual factors influencing job satisfaction are primarily personal values, but also personality and mental health [42]. 

Satisfaction with work is one of the main indicators of the quality of working life, taking into account its impact on the attitude of employees to work, satisfaction of specific needs of employees [43]. An individual who can talk to his or her colleagues and exchange not only work-related information, but also inquiries about well-being or polite conversation, builds a bond within the team. In turn, the individual has a sense of support and a friendly working environment, which contributes to a positive perception of work.

Studies to date show that social interaction has an important role to play in shaping job satisfaction [44]. When work is done remotely during the constraints of the COVID-19 pandemic, employees feel not only physically isolated but also psychologically isolated. Psychological isolation includes feeling a sense of emotional unfulfillment as a result of insufficient social contact and support [45]. Hence, in the presented analysis of factors influencing job satisfaction, the aspect concerning mutual relations at work was also considered.

The concept of employee relations (ER) refers to positive relations between two or more persons involved in a social and authoritative relationship in an organizational context [46].Workers who do the same work, by interacting and communicating with each other, tend to develop the same feelings about certain elements of the work or working conditions. Positive attitudes or frustration and dissatisfaction with work result from the degree to which needs related to work are fulfilled or satisfied [47]. Employees’ expectations are formulated on the basis of the information obtained regarding the tasks performed. According to the EST theory, the group members use the attributes of others to formulate their expectations of performance [24]. Positive ER in the workplace are characterized by high quality interaction between employees and supervisors and a sense of community within the organization [48]. Thanks to the high quality of the ER, the employee has the information resources that are essential for the formulation of his or her expectations. Referring to the EST theory, this study predicts that the ER as part of a wellbeing working environment will lead to an increased sense of job satisfaction. Therefore, this study assumes that there is a link between employee relations and job satisfaction. Hence we propose the following hypothesis:

**Hypothesis** **1** **(H1):** *Employees’ relations positively affect job satisfaction.*

### 2.3. The Mediating Role of Trust in Building Job Satisfaction 

Trust is a pillar of many areas and processes of organization functioning [49,50]. In an organizational context, trust is defined as the mutual relationship between two or more parties [51]. Trust is considered to be a ‘social bond’ necessary to maintain different organizational structures [52]. The literature stresses that organizational trust plays a very important role in the functioning of any organization [53,54]. 

Organizational trust can be analyzed in the inter-organizational and intra-organizational dimension [55]. In inter-organisational trust studies, the focus is on trust between entities, for example in the supply chain or network of organisations [56]. Intra-organizational trust focuses on relationships at the workplace. It concerns both mutual relations between employees and relations between employees and their supervisors [57,58,59].

A specific type of intra-organizational trust is interpersonal trust. Interpersonal trust pertains to the relationship between members of an organization [53,55]. Thanks to the trust in colleagues and emotional bonds between them, the employee knows that he or she can count on help/support in solving all kinds of problems, including those related to remote work during the pandemic period [60]. Employee relations are an important support in the perception of the work environment and the situation of an individual at work.

As both vertical [61,62] and horizontal [63] relationships in the workplace can have an impact on job satisfaction, our research encompasses both trust in co-workers and trust in the employee-manager relationship.

In an organizational context, trust is an effective predictor of positive attitudes and behavior of employees [64]. The results of previous research indicate that interpersonal trust positively influences the effectiveness and performance within an organization [65], commitment [66], collaboration [67], improving organisational loyalty [68], resistance to change and reduced stress levels [69] and a decrease in employee turnover [70]. In addition, trust activates the learning process by generating social relationships using different communication channels [71]. In an environment of a high level of trust, a safe working climate is created, employees build long-term relationships, and thus cooperation is strengthened [72]. In organizations with a high level of mutual trust, employees participate in decision-making processes, feel happy coming to work, are more creative in performing their tasks [73,74]. In summary, interpersonal trust determines and improves management processes, influences the nature of interpersonal relationships [75] and job satisfaction [68]. These factors may contribute to employees’ resilience to high levels of uncertainty and stress caused by pandemic and remote work [76]. Hence, it seems that it is the interpersonal trust in the organization that can indirectly strengthen the mental wellbeing of employees based on positive job evaluation.

Interpersonal trust is a dynamic and continuous process, which is based on mutual relations and taking actions to build trust in the organization [77]. An organization based on trust is an organization that strives to achieve success by actively involving employees [68]. Trust-supporting work environment, sense of community within the organization helps to build high quality employee relations [48]. Mutual trust is the basis of good relations in the workplace as well as one of the factors influencing job satisfaction. Hence we hypothesise that:

**Hypothesis** **2** **(H2):** *Interpersonal trust mediate the relationship between employee relations and job satisfaction.*

In this study, the relationships of the different forms of trust within organizations will also be analyzed: trust in a co-worker and trust in managers in order to illustrate more precisely the mechanisms explaining the analyzed relationships. Therefore, additional partial hypotheses have been derived.

Previous research indicates that trust-based coworkers’ relationship affects job satisfaction [78]. Moreover, the previous studies indicate the relationship of trust between employees and job satisfaction [79]. The importance of trust is growing especially in a virtual environment. Collaboration in remote teams is closely linked to the cognitive, social and emotional challenges faced by such teams [80]. During the pandemic, for many employees, working remotely and collaborating in a virtual team was a new challenge and first-time experience [18].

Building a high level of trust is essential to increase the satisfaction of remote working [81] and the performance of virtual teams [82]. Based on the perception of interpersonal trust, team members develop a common climate of trust when the views of team members converge [83]. Moreover, participation in leadership by promoting trust increases satisfaction in virtual teams both directly and indirectly [84]. The satisfaction of a team directly contributes to the satisfaction of its members, and the increase in individual satisfaction results in an increase in overall team satisfaction [84]. Trust also plays an important role in moderating the relationship between team members’ perception of the virtual effective use of communication and team performance [85]. Further, the impact of trust in colleagues on teamwork satisfaction has been indicated [86]. In view of the data presented above, the following hypothesis can been formulated:

**Hypothesis** **H2a** **(H2a):** *Interpersonal trust in colleagues mediates the relationship between employee relations and job satisfaction.*

In a similar vein, empirical data demonstrate that confidence in managers is an important factor contributing to job satisfaction [62]. Trust in supervisors affects employees’ sense of satisfaction [87]. Also in the situation of remote work and virtual teams, trust in the e-leader influences job satisfaction [88]. This means that trust in managers has a significant role to play in shaping job satisfaction. Therefore, a second partial hypothesis was derived, assuming that:

**Hypothesis** **H2b** **(H2b):** *Interpersonal trust in managers mediates the relationship between employee relations and job satisfaction.*

To sum up, this study examines the relationship of employee relationships to job satisfaction, based on a parallel mediation model that integrates the roles of interpersonal trust in colleagues and managers.

### 2.4. Methods

#### 2.4.1. Research Context

The increase in employment in remote form so far has been dictated by many factors. Most often, companies looking for qualified professionals offer remote working to attract employees regardless of their geographical location [80,89]. Such companies include those in the information technology (IT) sector. The IT sector includes companies that produce software, hardware or provide Internet services [90]. Revenues of IT companies operating in Poland in 2019. accounted for PLN 68 billion, or 61.3% of the revenue of the entire ICT (Information and Communication Technology) sector [91]. 3.1% of all employees in Poland are employed in the ICT sector, compared to 3.9% in the EU [92]. Virtual teamwork became a routine part of professional activity both in the IT and other sectors [93]. During the COVID-19 pandemic workers from many sectors were additionally directed to remote working.

In addition to the stress of the global health situation, remote workers have had to overcome the difficulties associated with remote working. In this case, remote working was not the form of flexible employment that the worker needed or expressed a desire for [85]. As previous studies have shown, some employers understand the difference between an office worker and a virtual employee only as a lack of physical presence, resulting in a less effective employee-employer relationship [94]. In fact, the differences between office workers and remote workers are immense in terms of how individuals can cooperate, collaborate, access information and contribute to the creation of knowledge [94], which is why it is so important to ensure proper relations between workers working virtually.

In addition, pandemics such as COVID-19 have adverse consequences for mental health [95], as workers are under additional stress for their own and their families’ health. During a pandemic, it is difficult to maintain psychological resilience, which is generally defined as the ability to maintain or regenerate mental well-being during or after a stressful period [96]. Understanding the mechanisms that support the positive mental health of employees, including job satisfaction, is vital in contemporary organizations.

#### 2.4.2. Instrument

The variables and scales used in these studies were subsequently selected on the basis of a review of existing literature. All questions have been asked using the five-point Likert scale, where 1 means “I strongly disagree” and 5 is defined as “I strongly agree”. Such a scale is considered appropriate for the study of the perception of workers in scientific research [97]. The study employs instruments that have been empirically validated in earlier studies.

The independent variable in this study is “employee relations”. It consisted of the following questions: “I have a good relationship with my co-workers”; “I have a sense of mutual support in my organization”; “I perceive my organization as a community”, “In my organization, regardless of the position in the structure, there are friendly relations between employees”, “There are good relations between employees in my organization”; “I consider the relationship between employees in my organization to be definitely positive”. This measure was constructed on the basis of the measure used by Bulińska-Stangrecka and Bagieńska [98] in the employee investigation in the context of innovation.

The dependent variable has been established as „job satisfaction”. The variable consists of three items: “I am satisfied with my position in the team”; “My work is satisfactory”; “I feel satisfied with my role in the organization”. The question was based on Jiang et al. [99].

The mediating variables used in the study include: “trust in managers” and “trust in colleagues”. The trust in managers contained four elements: “I have trust in the management of my organization”; “I could allow the management to have full control over my future of this organization”; “I trust that the decisions taken by the management are beneficial to the organization”; “I would feel comfortable giving the management a task or problem that is critical to me, even if I could not monitor their actions”; based on Mayer and Davis [100]. The following elements were part of the variable “trust in colleagues”: “If I got into difficulties/complications at work, I know that my colleagues would try to help me”; “I can trust the people I work with to help me when I need it”; “I am convinced that my colleagues will always try to treat me fairly”; “Most of my colleagues can be relied upon to do as they say”; “I have full trust in the skills of my colleagues”. This variable has been created on the basis of a research instrument by Cook and Wall [101].

To ensure that there is no common method bias in the study, a collinearity analysis was carried out. The Variance Inflation Factor (VIF) value of any of the analyzed variable indicators did not exceed 2, which excluded the risk of common method bias [102].

#### 2.4.3. Sample and Data Collection

The data used in this study were obtained between April and June 2020, during the first wave of the COVID-19 pandemic. The questionnaire was tested in a pilot study with 11 IT professionals. As a result of the pilot study, several units were adapted to make them understandable for the respondents. The questionnaire was sent to the IT industry employees. The survey was addressed to people working in organizations that are on the list of 366 companies on the TOP200 list [91]. Organizations were sent e-mail inquiries and an electronic version of the questionnaire was sent directly to employees. The convenience sampling method was used. As pointed out in the literature, this is a commonly used method in research, due to the costs and difficulties arising from probability sampling [103]. 

All participants gave their permission to use the data for research. Their responses were anonymous and treated as confidential. The survey data were collected in MS Excel. Statistical analyses were performed using RStudio (Boston, MA, USA) [104].

### 2.5. Data Analysis

#### 2.5.1. Common Method Variance

Due to the possibility of common method bias arising from the self-reporting method of data collection, Harman’s single factor test was conducted [105]. First, Harman’s CFA single factor test was performed. The test results indicated that Harman’s CFA single factor model had a much worse fit than the trait model (Table 1). To confirm the robustness of the hypothesised model, tests of three alternative models were carried out. The other alternative models also showed significantly worse fit than the trait model. The analysis performed indicated that common method bias is not a serious problem in this study [105].

#### 2.5.2. Measurement Model Estimation

Further analysis was preceded by a rigorous analysis of the hypothesized model. The data analysis was started by carrying out confirmatory factor analysis [CFA] to verify and validate the structure of the factors used in the model. Model fit was again rigorously tested before undertaking further analysis. Final measurement model indices were: CMIN/DF = 2.221, RMSEA = 0.074, CFI = 0.941 and TLI = 0.929 which indicated a good fit [107,108]. These indicators are summarized in Table 2.

Table 3 shows the results of CFA. The analysis did not reveal convergent and discriminant validity issues. Reliability was measured by composite reliability [CR], and these were all above the recommended threshold of 0.70 [107]. 

An analysis of the model has been carried out in terms of discriminant validity, reliability and convergent validity. Table 3 and Table 4 presents the Cronbach’s alpha, composite reliability, average variance extracted (AVE), and square root of the AVE, as well as the correlations between the constructs. A Cronbach alpha of greater than 0.7 is considered acceptable [107]. The analysis showed (Table 3) that the Cornbach’s alpha coefficient of the analysed constructs is higher than 0.7 and therefore it is assumed that this confirms the internal consistency and reliability of the measures.

Convergent reliability was measured by composite reliability [CR], and these were all above the recommended threshold of 0.70 [107]. According to Hair [107], AVE should be 0.5 or greater to suggest adequate convergent validity. The measures under analysis meet the above criteria. Discriminant validity statistics for the constructs involved a comparison of the AVE and squared correlation between constructs (Table 4).

The represented results well above the cut-off value, thus confirming the internal consistency reliability and convergent validity of those constructs.

The next step of the analysis was to verify the research hypotheses on the basis of the Hayes [109] PROCESS procedure. The analysis was carried out in the R study program. The PROCESS macro [110] is a tool for advanced mediation analysis. It allows obtaining simultaneous direct, indirect and total effect estimators and assessing the associations between them [110]. The use of PROCESS makes it possible to generate bootstrapped confidence intervals to prevent errors in calculations.

It is assumed that if the confidence intervals do not contain zero, the test is statistically significant [109]. The test is a reliable tool for verifying the effects of mediation and produces more accurate results than the Sobel test [111].

The bootstrapping approach with 1000 bootstrapping samples was used. In line with this approach, the indirect effects of interpersonal trust as mediators of relations between employee relations and job satisfaction were analyzed. Both the total, indirect and direct effects have been examined. The analysis of 95% confidence intervals (CI) was used as an indicator of statistical significance. Where the interval between low (LLCI) and high (ULCI) is zero, the mediation result is considered not to be statistically significant. The assumption was made for partial mediation in this study. The occurrence of partial mediation refers to the analysis where the indirect effect βyx.m does not fall below zero and where mediation (indirect effect of X on Y) is statistically significant (p level).

## 3. Results

### 3.1. Descriptive Statistics 

The profile of respondents is presented in Table 5. Most of the respondents were men (77.3 percent). This reflects EU data on female employment in the IT industry [92]. Average time of professional experience in the IT industry among the respondents is 9.99 years (min <1 year, max 43 years). More than half (55 per cent) of the survey participants have a master’s or university degree. By far the largest number of respondents work in a specialist position (62.7 percent), while 15 percent work as managers and 10 percent as directors. Each person who completed the survey questionnaire was employed in the IT industry.

### 3.2. Correlation among Variables 

The analysis of the correlation between the investigated variables is presented in Table 6. The results showed that there are positive and significant correlations between the trust in managers, trust in colleagues, job satisfaction and employee relations, which indicates that further data examination can be carried out.

### 3.3. Hypothesis Testing

Empirical verification of hypothesis H1 (Employee relations positively affect job satisfaction) based on linear regression analysis indicates that employee relationships positively affect job satisfaction (β = 0.367; (F(1,218) = 27.1; *p* < 0.001), and explained 11 percent of variance (R^2^ = 0.11). 

### 3.4. Mediation Analysis 

The mediation analysis results are described in Table 7. The obtained data confirmed that the total effect was statistically significant (βyx = 0.366; LLCI = 0.227; ULCI = 0.505; *p* < 0.001). Additionally, when the mediation effect was added, while controlling the independent variable (X Employee relations), the total effect was still statistically significant, but its value was reduced: βyx.m = 0.167; LLCI = 0.017; ULCI = 0.316. The model explains 9 percent of the variance in job satisfaction. Significant result was shown by the analysis of the ratio of indirect to total effect of X on Y: β = 0.544, LLCI = 0.292; ULCI = 0.968.

**Figure 1:**
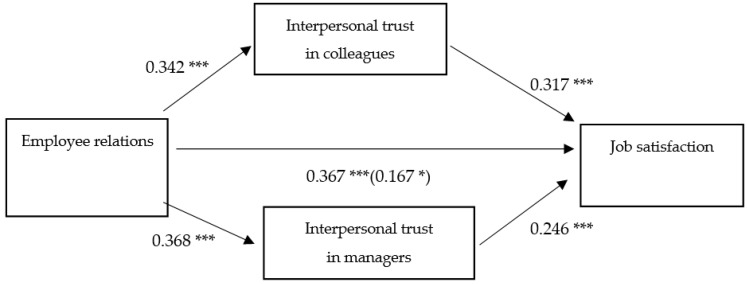
Parallel mediation model (*n* = 220). Indirect effects of employee relations on job satisfaction through interpersonal trust in colleagues and interpersonal trust in managers. Standardized effects estimates are presented. The effects on the direct path from employee relations to Job satisfaction depict the direct effect and the (total effect). * *p* = 0.028, *** *p* < 0.001.

Results based on 10,000 bootstrapped samples indicated that the total effect of employee relations on job satisfaction was significant (β total = −0.367, SE = 0.070, *p* < 0.001), the direct (β direct = −0.0167, SE = 0.075, *p* = 0.258) and indirect effects are present (Figure 1).

The ratio of interpersonal trust to colleagues and interpersonal trust to managers to the total effect is β = 0.296 LLCI = 0.122; ULCI = 0.599 and β = 0.248 LLCI = 0.108; ULCI = 0.520 respectively.

Results from a parallel mediation analysis indicated that employee relation is indirectly liked to job satisfaction through its relationship with interpersonal trust to managers and interpersonal trust to colleagues.

The results of the presented analysis confirm that the positive relationship between employee relations and job satisfaction is partly dependent on trust in managers and trust in co-workers. Thus, the above results are evidence of positive H1: Employees relations positively affects job satisfaction verification. Moreover, the mediation results confirm the H2 hypothesis (Interpersonal trust mediates the relationship between employee relations and job satisfaction). Additionally, individual analyses made it possible to support partial hypotheses H2a (Interpersonal trust in colleagues mediates the relationship between employee relations and job satisfaction) and H2b (Interpersonal trust in managers mediates the relationship between employee relations and job satisfaction) and demonstrate the role of intra-organizational trust in shaping job satisfaction. The results are presented in Table 8.

The aforementioned results support the assumptions about the positive impact of employee relations on job satisfaction. In addition, this study demonstrates that the relationship is partly mediated by inter-organizational trust: including both trust in managers and trust in colleagues.

## 4. Discussion

The main objective of these research is to examine the relationship between employee relations and job satisfaction and interpersonal trust as perceived by employees from the IT industry in Poland. Results from the mediation analysis have proven that employee relations indeed have a significant and positive relationship with job satisfaction and this link is mediated by trust in managers and trust in colleagues among Polish IT companies surveyed. 

The verification of the hypotheses showed that employee relations have a significant positive impact on job satisfaction. The verification of the hypotheses demonstrated that employee relations have a significant positive impact on job satisfaction. Additionally, interpersonal trust in both colleagues and managers is a significant factor in explaining this effect. By enhancing the positive anticipation of group members’ actions, trust reinforces positive expectations of the group and leads to attitudinal adjustments that have a positive impact on the well-being of employees.

These findings are consistent with Güçer and Demirdağ [68], where the level of organizational trust perception affects job satisfaction of the hotel employees. Furthermore, the results are in accordance with the findings of Dimotakis et al. [44], implying that interpersonal interactions affect job satisfaction. This study implies that positive employee relations stimulate perceived job satisfaction through mediated effects of interpersonal trust. Hence, it is crucial for managers to ensure that positive interaction between employees is fostered in the organization and a climate of mutual trust is established. 

This study shows that trust in both employees and managers has an impact on the well-being of employees. In the context of the well-being of employees based on perceived information according to EST, this is an important indication of the assumptions that determine the formation of expectations towards fellow employees. Therefore, this study indicates that the better the relationship with colleagues the higher the job satisfaction. Moreover, interpersonal trust explains the interplay of this relationship and demonstrates the significant impact of trust in colleagues and managers in shaping positive well-being at work.

According to the theory, the formation of job satisfaction is based on the perceived relationships within the organization, which justifies how important it is to form positive relationships between employees [16]. Additionally, it stresses that inter-organizational trust is an important factor strengthening the above described relations between employees and job satisfaction. In conclusion, interpersonal trust is a key driver of job satisfaction among the surveyed employees.

Furthermore, the significance of trust within the organization increases in the context of remote working. This is in line with the Flavian et al. [88] results, which demonstrate how trust shapes the foundations of cooperation in virtual teams. The changes resulting from the geographical dispersion which is the consequence of remote working need to promote trust between workers. This is in line with El-Kassrawy [112], where the trust has been recognized as having a significant impact on satisfaction in virtual teams. Hence, this study also highlights the role of employee relations based on interpersonal trust. From this perspective, it is important to enable employees to build positive relationships based on interaction and mutual trust, through the use of available tools (in particular means of electronic communication) to ensure the remote employee’s well-being in a pandemic situation.

## 5. Conclusions

In the light of the COVID-19 pandemic, research on the mental health of workers is becoming increasingly important. Due to various factors negatively affecting the well-being of workers, such as the fear of illness or social isolation, it is becoming extremely important to maintain healthy relationships at a remote workplace. Understanding the mechanisms that increase job satisfaction and contribute to the improvement of mental well-being will enable managers to take appropriate measures to create a friendly working environment.

EST has been discussed in this study in the context of shaping behavioral patterns based on expectations towards colleagues. Through the lens of EST theory, the relationship between working relationships, interpersonal trust and job satisfaction in the context of mental well-being was examined.

The results of this study integrate two streams of research: those concerning the relationship between expectations and their role in the functioning of the individual in the organisation (concerning EST theory) and those related to mental health at work, more specifically the analysis of the determinants of employee well-being.

In essence, this study points to a new research perspective on employee mental health embedded in dynamic group processes based on interpersonal trust and employee relations.

The potential repercussions of failing to recognize the significance of employee relations and interpersonal trust may include a deterioration in the mental well-being of employees. It is therefore important to allow employees to interact.

These studies highlight the key role of employee relations in building job satisfaction. They indicate how important it is to provide virtual space for establishing and maintaining social relations remotely. Moreover, these studies illustrate how important interpersonal trust is in supporting the relationship between employees and job satisfaction. By increasing interpersonal trust, the potential of social relationships in remote working conditions is strengthened. This study focuses on both trust in managers and colleagues, demonstrating the mechanisms that facilitate the mental well-being of employees in remote working situations in the time of the pandemic. From the results of these studies it can be established that employee relations built on trust are indeed the foundation of a supportive remote working environment.

Remote working can contribute to employee isolation by limiting interactions, especially those concerning non-work issues. In a remote working situation, there are fewer opportunities to ask colleagues about their wellbeing and to build social bonds at work. This may contribute to depression or deterioration of mental health. Our research indicates that employee relations and interpersonal trust are the driving forces behind job satisfaction. Therefore, it is important to focus not only on performance, but also on providing space for interaction.

## 6. Limitations and Further Studies

This study has, like any other research, some limitations. The first limitation is that it is a cross-sectional study, and therefore there are some constraints in terms of narrowing the time horizon of the study [113]. In order to strengthen the results of the studies, longitudinal studies should be carried out in the future. Another limitation concerns the geographical narrowing of the research that has been carried out in Poland. In order to confirm the results, future research should cover other countries. An additional limitation is also the restriction of the research to the IT sector. Further analyses should be extended to other sectors. Future studies should examine what role vaccines will play in the context of workers’ mental health. It is also important to explore the impact of other threats [114], on workers’ mental well-being in the light of the presented links.

## Figures and Tables

**Table 1:** Comparison of fit of the hypothesized model with alternative models.

Model	χ^2^	df	χ^2/df^	Δχ^2^	Δdf	CFI	RMSEA
Hypothesized model: four factor model	251	113	2.221			0.941	0.074
Alternative model 1: one-factor model (Harman’s one factor model)	1245	229	5.436	994	116	0.522	0.207
Alternative model 2: two-factor model (trust in managers and trust in colleagues, and job satisfaction and employee relations were combined	707	118	5.991	456	5	0.750	0.151
Alternative model 3: three-factor model (trust in managers and trust in colleagues were combined)	442	116	3.810	191	3	0.861	0.113

Note: All models are compared to the hypothesized model. df- degree of freedom; CFI- comparative fit index; RMSEA- The Root Mean Square Error of Approximation. Job satisfaction was used as a dependent variable to identify the model [106].

**Table 2:** Final measurement model fit indices.

Fit Index	Recommended Criteria	Authors	Results
CMIN/DF	<5	[107]	2.221
RMSEA	<0.8	[108]	0.074
CFI	0.9	[107]	0.941
TLI	0.9	[107]	0.929

Note: CMIN/DF: Minimum of Discrepancy/Degrees of Freedom; RMSEA: Root Mean Square Error of Approximation; CFI: Comparative Fit Index; TLI: Tucker–Lewis index.

**Table 3:** Reliability analysis of variables.

Measures	Construct Items	Estimate	SE	z	*p*-Value	Composite Reliability	Average Variance Extracted	Cronbach’s Alpha
Interpersonal trust in managers	ITM 1	0.838	0.0593	14.12	<0.001	0.896	0.7435	0.780
	ITM 2	0.669	0.0818	8.18	<0.001			
	ITM 3	0.759	0.0581	13.07	<0.001			
	ITM 4	0.709	0.0848	8.36	<0.001			
Interpersonal trust in colleagues	ITC1	0.755	0.0498	15.15	<0.001	0.883	0.607	0.854
	ITC1	0.767	0.0557	13.79	<0.001			
	ITC1	0.802	0.0572	14.01	<0.001			
	ITC1	0.927	0.0572	16.20	<0.001			
	ITC1	0.612	0.0750	8.17	<0.001			
Job satisfaction	JS1	0.817	0.0558	14.64	<0.001	0.769	0.532	0.810
	JS2	0.562	0.0579	9.69	<0.001			
	JS3	0.785	0.0544	14.44	<0.001			
Employee relations	ER1	0.838	0.0537	15.59	<0.001	0.943	0.806	0.935
	ER2	0.938	0.0572	16.42	<0.001			
	ER3	0.986	0.0569	17.33	<0.001			
	ER4	0.861	0.0517	16.64	<0.001			
	ER5	0.857	0.0625	13.72	<0.001			

The construct items are used to explain the construct.

**Table 4:** Correlations between constructs.

	AVE	1	2	3	4
1. Interpersonal trust in managers	0.743	0.861 *			
2. Interpersonal trust in colleagues	0.607	0.188	0.779 *		
3. Job satisfaction	0.532	0.359	0.343	0.729 *	
4. Employee relations	0.806	0.306	0.436	0.332	0.897 *

Notes: * The bold number is the square root of AVE. The bold numbers listed diagonally are the square root of the variance shared between the constructs and their measures. The off-diagonal elements are the correlations among the constructs. For discriminate validity, the diagonal elements should be larger than the off-diagonal elements.

**Table 5:** Demographic profile of respondents.

Variable	Characteristics	N	%
Gender	Female	50	22.7
	Male	170	77.3
Education	Master Degree or higher	121	55
	Engineering Degree	56	25.5
	Bachelor Degree	12	5.5
	High school and studying	31	14.1
	CEO, board member	11	5
Position	Director	22	10
	Manager	33	15
	Specialist	138	62.7
	Assistant	16	7.3
Company size	>250	107	48.6
	<250	53	24.1
	<50	45	20.5
	<10	15	6.8

**Table 6:** Pearson correlation coefficients between study variables.

	TM		TC		JS		ER		Size		Position		Education		Gender	Experience
Trust Managers	—															
Trust Colleagues	0.188	**	—													
Satisfaction	0.359	***	0.343	***	—											
Relations	0.306	***	0.436	***	0.332	***	—									
Size	−0.152	*	0.027		−0.127		−0.042		—							
Position	0.222	***	0.097		0.392	***	0.167	*	−0.306	***	—					
Education	−0.069		−0.062		0.054		0.074		0.098		0.127		—			
Gender	0.136	*	0.011		0.016		−0.006		−0.003		−0.039		0.054		—	
Experience	0.005		−0.062		0.225	***	−0.008		−0.148	*	0.606	***	0.154	*	−0.092	—

Note. * *p* < 0.05, ** *p* < 0.01, *** *p* < 0.001.

**Table 7:** Total, direct and indirect links between employee relations and job satisfaction through psychological trust in managers and trust in colleagues.

				Bootstrap 95% Confidence Interval (CI)	
Effect [β]	SE	t	*p*	LLCI	ULCI
Total effect (βyx): Employee relations (X) on job satisfaction (Y)					
0.367 F_p_ = 27.091 *** R^2^ = 0.110	0.070	5.204	<0.001	0.227	0.505
Direct effect: Employee relations (X) on job satisfaction (Y)					
0.167	0.075	2.201	0.028	0.017	0.316
Indirect effect (βyx.m) Employee relations (X) on job satisfaction (Y) through the mediating variables (M)					
Interpersonal trust in managers					
0.091	0.029			0.042	0.163
Interpersonal trust in colleagues					
0.108	0.039			0.045	0.204

Notes: lower level confidence interval (LLCI); upper level confidence interval (ULCI). Number of bootstrap samples for bias corrected bootstrap confidence intervals: 10,000. Level of confidence for all confidence intervals in output: 95%. N = 220, *** *p* < 0.001.

**Table 8:** Results of hypothesis testing.

Hypotheses	Results
**H1**: *Employee relations positively affect job satisfaction*	Supported
**H2**: *Interpersonal trust mediates the relationship between employee relations and job satisfaction*	Supported
**H2a**: *Interpersonal trust in colleagues mediates the relationship between employee relations and job satisfaction*	Supported
**H2b**: *Interpersonal trust in managers mediates the relationship between employee relations and job satisfaction*	Supported

## Data Availability

The data presented in this study are available on request from the corresponding author.
